# Sustainable Approach to Thermoplastic Polyurethanes Stabilization: Vanillin as a Natural Additive in Melt-Processing

**DOI:** 10.3390/polym18202539

**Published:** 2026-10-16

**Authors:** Joanna Smorawska-Kliza, Anna Jażdżewska, Ewa Głowińska

**Affiliations:** Department of Polymer Technology, Faculty of Chemistry, Gdańsk University of Technology, 11/12 Gabriela Narutowicza Street, 80-233 Gdańsk, Poland; joanna.smorawska@pg.edu.pl (J.S.-K.); s189238@student.pg.edu.pl (A.J.)

**Keywords:** vanillin, free-radical scavenger, high-temperature processing, bio-based thermoplastic polyurethanes, sustainable development, environmental impact

## Abstract

Thermo-oxidative degradation during high-temperature processing remains a major challenge limiting the performance of thermoplastic polyurethanes (TPUs). This study evaluated vanillin (4-hydroxy-3-methoxybenzaldehyde), a bio-based phenolic compound, as a primary thermo-oxidative stabilizer acting as a free-radical scavenger. Bio-based TPU (bio-TPU) was synthesized via a two-step method using a commercial bio-based polyol, a bio-based chain extender, and a mixture of diisocyanates. During processing in an internal mixer, vanillin was incorporated into both bio-TPU and a commercial TPU at concentrations of 0.03, 0.165, and 0.3 wt%. Changes in chemical structure were assessed by Fourier-transform infrared spectroscopy (FTIR), thermal properties were evaluated by thermogravimetric analysis (TGA) and differential scanning calorimetry (DSC), including oxidation onset temperature (OOT) measurements, and dynamic mechanical analysis (DMA). Mechanical properties were assessed by tensile test and hardness. FTIR analysis revealed no significant structural changes after vanillin addition. However, changes in thermal properties were observed, including an increase in the OOT for materials containing the highest vanillin content. TGA results also showed a generally improved thermal stability at higher vanillin concentrations, approaching reference-material values at the highest concentration in the commercial TPU. A slight decrease in mechanical properties was observed. These findings demonstrate a pronounced stabilizing effect of 0.3 wt% vanillin on both bio-TPU and commercial TPU, providing new insights into the potential reuse and further recycling of TPU materials stabilized with bio-based additives.

## 1. Introduction

Material degradation is a widely recognized challenge for both researchers and manufacturers of polymer-based products. In the context of materials science, degradation refers to changes occurring in a material as a result of exposure to abiotic factors, such as temperature, oxygen, or UV radiation, as well as biotic factors, including different microorganisms, such as bacteria and fungi [1,2]. Regardless of the degradation factor, the process generally results in the deterioration of material properties and often in the loss of structural integrity compared to the original material.

Degradation of polymer-based products may contribute to the release of microplastics into the environment and their subsequent accumulation. Microplastics can even be further degraded to nanoplastics [3]. Polymeric materials are exposed to degradation processes not only during their daily use, but also during processing, post-processing, and product forming. Degradation during processing may result from elevated temperature, the presence of oxygen, and shear forces, which accelerate thermo-mechanical and thermo-oxidative reactions [4]. Among degradation processes, photo-oxidation (limited to the material’s surface) and thermo-oxidation (occurring throughout the bulk material, particularly critical during processing) are the most significant.

Thermoplastic polyurethanes (TPUs) constitute an important group of polymeric materials, valued for their tunable chemical structure and, consequently, their versatile properties, as well as their processability via conventional methods such as injection molding and extrusion, enabling numerous applications, including tubes, films, protective cases, and watch straps [5,6]. Such products may differ not only in chemical structure but also in service conditions. Therefore, degradation in the context of polyurethanes is a complex process and is primarily associated with thermo-oxidative degradation, which involves the simultaneous effects of elevated temperature and oxygen [7]. Delaying degradation and stabilizing TPU materials are of considerable importance. Owing to their thermoplastic nature, TPUs can be recycled using various methods, including mechanical recycling, whereby post-industrial TPU waste can be reintroduced into the processing cycle. However, degradation may cause structural changes and thereby lead to the deterioration of processing properties of the material and, consequently, may hinder or prevent its reuse in subsequent processing steps. Thermo-oxidative degradation of TPU proceeds as a chain of radical reactions occurring within the polymer chain. In the first stage, macroradicals may be formed and subsequently react with oxygen present in the surrounding environment. Next, unstable peroxy radicals and hydroperoxides are generated and decompose further, causing degradation of the material [8].

Degradation affects both hard and soft segments of TPU. Urethane bond cleavage can generate isocyanate-related fragments, and studies on commercial TPUs confirm that multiple volatile degradation products form under thermo-oxidative conditions, indicating a multi-pathway mechanism [7]. Susceptibility also depends on soft-segment chemistry: polyether-based TPUs are prone to oxidation at carbons near ether linkages, while polyester-based TPUs, though more thermally resistant, are more susceptible to hydrolytic degradation of ester bonds [9].

To counteract degradation, commercially available polymers are typically formulated with functional additives, most notably antioxidants and thermal stabilizers. Antioxidants are divided into primary antioxidants (radical scavengers, mainly sterically hindered phenols (ArOH) such as BHT and the Irganox series) and secondary antioxidants (hydroperoxide decomposers, mainly phosphites and phosphonites such as the Irgafos series, and thioethers such as DLTDP, DSTDP, DMTDP). Phosphorus-based secondary antioxidants are more thermally stable and are used during processing, while thioethers are better suited for long-term stabilization; the two classes may also act synergistically.

Natural antioxidants—tocopherols, rosemary and green tea extracts, flavonoids, lignans, carotenoids, ascorbic acid, and ferulic acid—have attracted growing interest [10,11]. Among these, only α-tocopherol has reached commercial application as a polymer stabilizer (Irganox^®^ E 201) [12], mainly studied in the case of polyolefins. Other natural compounds such as catechins, quercetin, and tannic acid have shown promise in bio-based polymers (PLA, starch, PHB) [13,14], but research remains largely fundamental, and synthetic antioxidants still dominate the industry. Growing safety concerns around conventional additives like BHT [15] are driving a shift toward natural or hybrid stabilizer systems that combine natural and synthetic antioxidants to balance performance and sustainability.

Vanillin (4-hydroxy-3-methoxybenzaldehyde), a bio-based phenolic aldehyde derived from vanilla or lignin depolymerization, is a promising candidate in this context. In general, vanillin can be derived from plants or chemically synthesized, and price depends on its origin [16]. In the context of the antioxidant activity, its phenolic hydroxyl group, activated by an adjacent electron-donating methoxy group, allows vanillin to act as a hydrogen-donating primary antioxidant, analogous to hindered phenols [17,18]. Vanillin has also been reported to impart thermal stability and improve mechanical properties of polymers, and its industrial availability and GRAS (Generally Recognized as Safe) status further strengthen its use as a bio-based functional additive [19]. Notably, vanillin may constitute a precursor for polyurethane synthesis, e.g., vanillin-based polyols from vanillin and two sustainable feedstocks, 2,5-furandicarboxylic acid (FDCA) and terephthalic acid (TPA) [20], or vanillin diol (VAN-OH) based on vanillin and m-xylylenediamine (MXDA) containing a dynamic imine bond [19,21].

Bio-based TPUs (bio-TPUs) seek to replace petroleum-based feedstocks with plant-derived equivalents, principally bio-based polyols and bio-based chain extenders, while fully bio-based isocyanates remain a subject of active research. Several studies, including our previous research [22,23], have demonstrated that bio-TPUs can indeed achieve comparable performance to conventional TPUs. The novelty of this study lies in the intersection of two research directions outlined above: the stabilization of bio-based thermoplastic polyurethanes and the application of vanillin as a natural phenolic antioxidant in melt processing, which has not been described before. This study examines the influence of vanillin addition on synthesized bio-TPU and commercial TPU, focusing on their structure, thermal, thermo-mechanical, and physical properties.

## 2. Materials and Methods

### 2.1. Materials

For the synthesis of bio-based thermoplastic polyurethane materials (bio-TPUs), 100% bio-based poly(trimethylene ether) glycol (PO3G) with an average molecular weight M_n_ of ~2000 g/mol, purchased from Sigma-Aldrich (St. Louis, MO, USA), was used as the polyol component. The isocyanate phase consisted of petrochemical hexamethylene diisocyanate (HDI, Vencorex, Saint-Priest, France) combined with partially bio-based (25% bio-based content) Tolonate™ X FLO 100 (Vencorex, Saint-Priest, France) in a weight ratio of 80:20. A total of 100% bio-based 1,3-propanediol (bio-PDO, Susterra^®^, DuPont Tate and Lyle Bio Products, Wilmington, DE, USA) was used as a chain extender. For comparison with the bio-TPU system, a polyether-based TPU Elastollan^®^ 1185A (BASF Polyurethanes GmbH, Lemförde, Germany) was employed. According to the literature, this commercial TPU is based on poly(tetramethylene ether) glycol (PTMG), methylene diphenyl diisocyanate (MDI), and 1,4-butanediol (BDO), and contains ~40.8 wt% hard segment [24]. Vanillin was purchased from Sigma-Aldrich (St. Louis, MO, USA).

### 2.2. Synthesis of Thermoplastic Bio-Polyurethane

**Figure 1:**
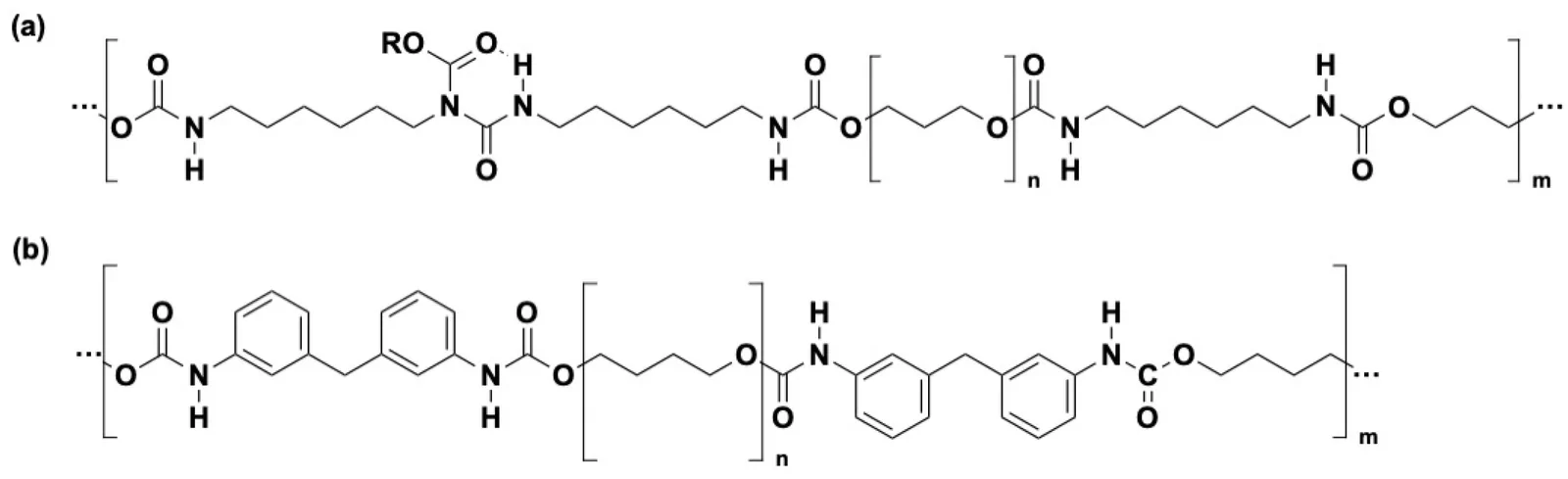
Proposed chemical structures of (**a**) the synthesized bio-TPU (HF) [25], based on aliphatic diisocyanates mixture (HDI/Tolonate^TM^ X FLO 100), and (**b**) the commercial TPU (E, Elastollan^®^ 1185A), based on an aromatic diisocyanate MDI [24].

Bio-based thermoplastic polyurethanes (bio-TPUs) were synthesized using a two-step prepolymerization method. In the first stage, the dried polyol was reacted with a calculated amount of the isocyanate mixture (HDI/Tolonate™ X FLO 100, 80:20 wt%). The reaction was carried out under controlled temperature conditions, resulting in the formation of an NCO-terminated prepolymer. In the second stage, bio-based 1,3-propanediol (bio-PDO) was added as a chain extender in an amount corresponding to an isocyanate-to-hydroxyl molar ratio ([NCO]/[OH]) of 0.95. The reaction mixture was intensively stirred until a homogeneous system was obtained. Subsequently, the reaction mixture was degassed under reduced pressure and poured into a steel mold preheated to 80 °C. After solidification, the samples were cured in a laboratory oven at 80 °C for 24 h to ensure completion of the polymerization process. The obtained materials were conditioned at room temperature prior to further melt processing. The resulting bio-TPU contained 31.0 wt% hard segments. The chemical structure of the obtained bio-TPU, as well as the commercial TPU, is visualized in Figure 1.

### 2.3. Processing

Vanillin was incorporated into the polymer matrix during melt processing as a natural stabilizing additive. Melt processing of the bio-TPU and the commercial TPU was performed using a closed internal mixer (Plasti-Corder Brabender GNF 106/2, Duisburg, Germany) equipped with a 50 cm^3^ mixing chamber, filled with 45 g of material per batch, under air atmosphere. The polymer was first plasticized under controlled temperature (150 °C for HF series and 180 °C for E series) at a rotor speed of 80 rpm. Vanillin was then added directly to the mixing chamber at concentrations of 0.03, 0.165, and 0.3 wt% relative to the polymer, and mixing continued for a total of 5 min per batch, until the additive was evenly distributed throughout the polymer matrix.

**Figure 2:**
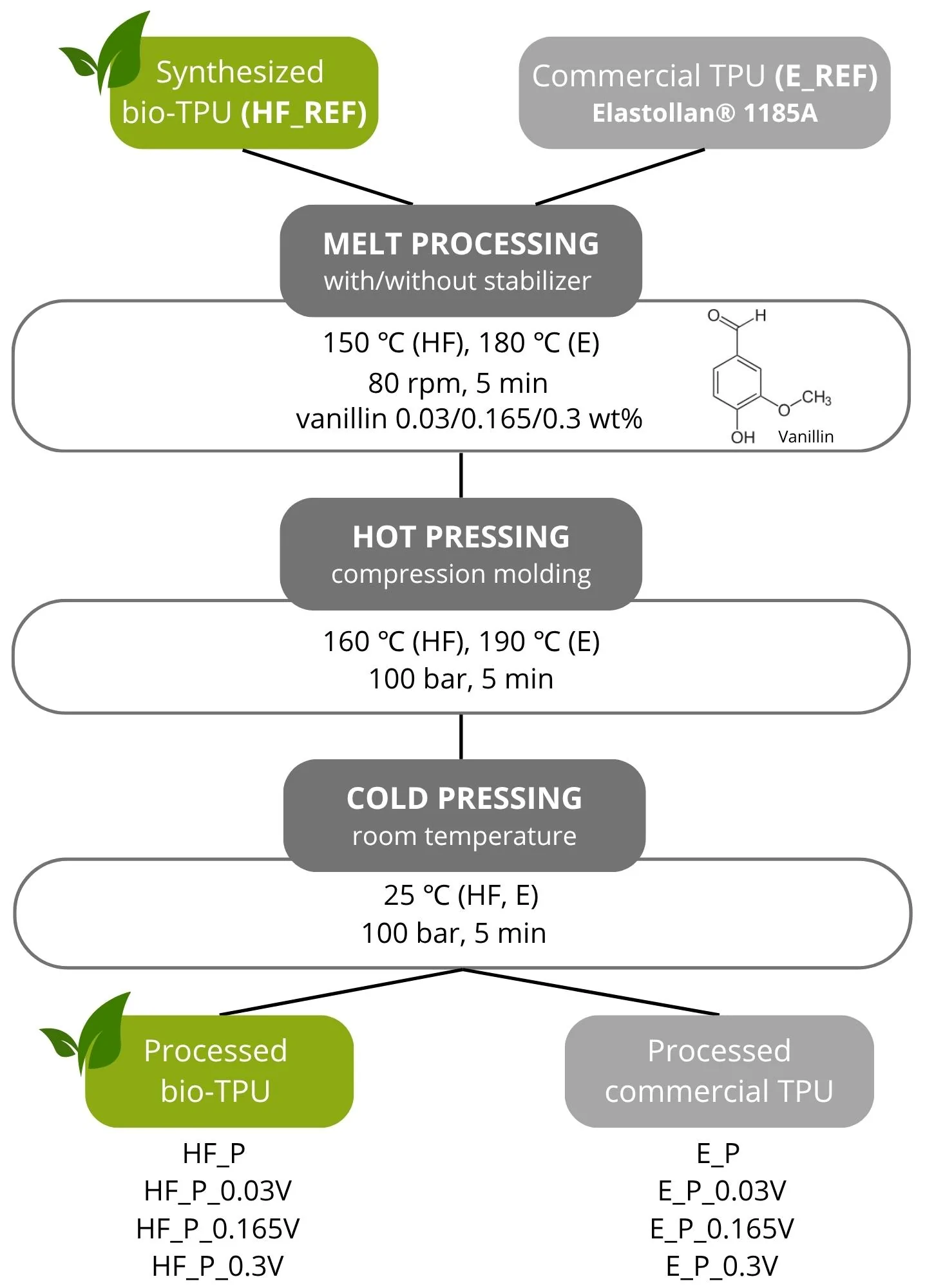
Schematic processing procedure of synthesized bio-TPU and commercial TPU.

The materials were then hot pressed for 5 min (at 160 °C for HF series and 190 °C for E series) and at 100 bar pressure. Cold pressing was performed as the final step at 25 °C and 100 bar for 5 min, to minimize the dimensional shrinkage of the samples. Schematic processing procedure was shown in Figure 2. The resulting sample series are summarized in Table 1 and the photographs of the obtained samples are provided in Table S1 in the Supplementary Materials.

### 2.4. Methodology

Fourier-transform infrared spectroscopy (FTIR) was used as a standard tool for investigation of the chemical structure of the synthesized bio-polyurethanes. Measurements were performed on an IRTracer-100 spectrometer (Shimadzu, Kyoto, Japan) equipped with an ATR (Attenuated Total Reflectance) accessory. Spectra were recorded in the wavenumber range of 500–4000 cm^−1^ with a resolution of 4 cm^−1^, accumulating 64 scans per sample. All measurements were carried out at room temperature.

Based on the FTIR results, deconvolution of the carbonyl band was performed. The spectra in the range of 1600–1800 cm^−1^ were normalized on the absorbance (Y) axis prior to deconvolution, and used to calculate the carbonyl index (R), degree of phase separation (DPS), and degree of phase mixing (DPM). Peak fitting was conducted using the Peak Analyzer function in the OriginLab software, which applies Gaussian peak-fitting functions. Baseline correction was carried out automatically by the software prior to fitting. During fitting, peak positions were not constrained; instead, the software determined the position, height, and width of each component peak based on the spectrum. The resulting positions were then compared with literature-reported values for hydrogen-bonded and free carbonyl groups to ensure they were chemically reasonable. The quality of each fit was assessed using the coefficient of determination (R^2^). R^2^ values ranged from 0.990 to 0.999 across all analyzed spectra. Detailed values for individual samples are provided in Table S2. Deconvolution was performed once per sample. The following Equations (1)–(3) were used for the calculations:

(1) $$R=\frac{A_{b}}{A_{f}}$$

(2) $$\mathrm{DPS}=\frac{R}{R+1}$$

(3) DPM = 1 − DPS

where A_b_ represents the combined peak heights (maximum absorbance) of the hydrogen-bonded carbonyl components, and A_f_ is the peak height (maximum absorbance) of the free (non-bonded) carbonyl component, as determined from the Gaussian deconvolution.

Thermogravimetric analysis (TGA) was conducted using a Pyris advanced thermal analysis system Perkin Elmer TGA800 (PerkinElmer, Waltham, MA, USA). Samples weighing ~10 mg were heated from 30 to 600 °C under a nitrogen atmosphere at a heating rate of 10 °C/min.

X-ray diffraction (XRD) was performed using a Philips X’Pert Pro diffractometer equipped with an X-ray tube with a copper anode, emitting CuKα radiation at a wavelength of 1.54 Å. The test was conducted with a 2θ range of 5–100°.

Differential scanning calorimetry (DSC) was carried out using a DSC 204F1 Phoenix calorimeter (NETZSCH, Selb, Germany) to examine the thermal transitions of the samples. Each sample, approximately 10 mg in weight, was placed in an aluminum crucible and analyzed under nitrogen. The measurement involved an initial heating cycle from −80 °C to 240 °C at a rate of 10 °C/min to erase thermal history, followed by cooling back to –80 °C at a rate of 5 °C/min. Finally, a second heating cycle from −80 °C to 240 °C at a rate of 10 °C/min was performed to determine the thermal properties of the materials.

The onset oxidation temperature (OOT) was also determined using the DSC apparatus. The samples were prepared in the same manner as described above and heated in an oxidative atmosphere (air) at a heating rate of 10 °C/min from room temperature to 300 °C. The OOT values were determined with the use of NETZSCH Proteus Thermal Analysis software v.8.0.2, using the onset function. Measurements were performed in triplicate (*n* = 3) for each sample, and results are reported as mean ± standard deviation.

Dynamic mechanical thermal analysis (DMTA) of bio-TPUs and commercial TPU was performed using a DMA Q800 analyzer (TA Instruments, USA). The test was conducted in single-cantilever bending mode. Specimens (10 mm × 40 mm) were heated from −100 °C to 150 °C at a rate of 3 °C/min, using a testing frequency of 1 Hz. Unlike the HF series, DMTA measurements for the E series could not be performed on the unprocessed reference material (E_REF), as this was supplied in pellet form and required melt processing and hot pressing to obtain specimens of suitable geometry for the DMTA test. Consequently, the E series data reflect only the effect of vanillin addition to the processed, unstabilized sample (E_P). That is why the effect of processing on the dynamic mechanical properties compared to the reference material can be evaluated only for HF series.

Tensile tests were performed using a standard Autograph AGS-X (Shimadzu, Kyoto, Japan) universal testing machine in accordance with PN-EN ISO 527-1 [26] and 527-2 [27] standards. Dumbbell-shaped specimens (type 5B) were tested at a crosshead speed of 200 mm/min. The initial distance between grips was set to 20 mm. Pre-load force was 1 N, with the pre-load speed of 5 mm/min. All measurements were performed at room temperature, and each sample was tested five times. Based on the tests, the tensile strength (T_Sb_) and nominal elongation at break (ε_b_) were calculated as the mean of five measurements.

Hardness of materials was evaluated using a Shore A durometer (Sauter, Balingen, Germany) according to PN-EN ISO 868:2005 [28]. The results are presented as the arithmetic mean of 10 measurements.

Statistical test, performed to assess significance of differences among samples within each series, was conducted using one-way ANOVA followed by Tukey’s post hoc test (α = 0.05), performed in OriginPro 2024. Measurements were prepared for the OOT, tensile strength, elongation at break, and hardness.

## 3. Results and Discussion

**Figure 3:**
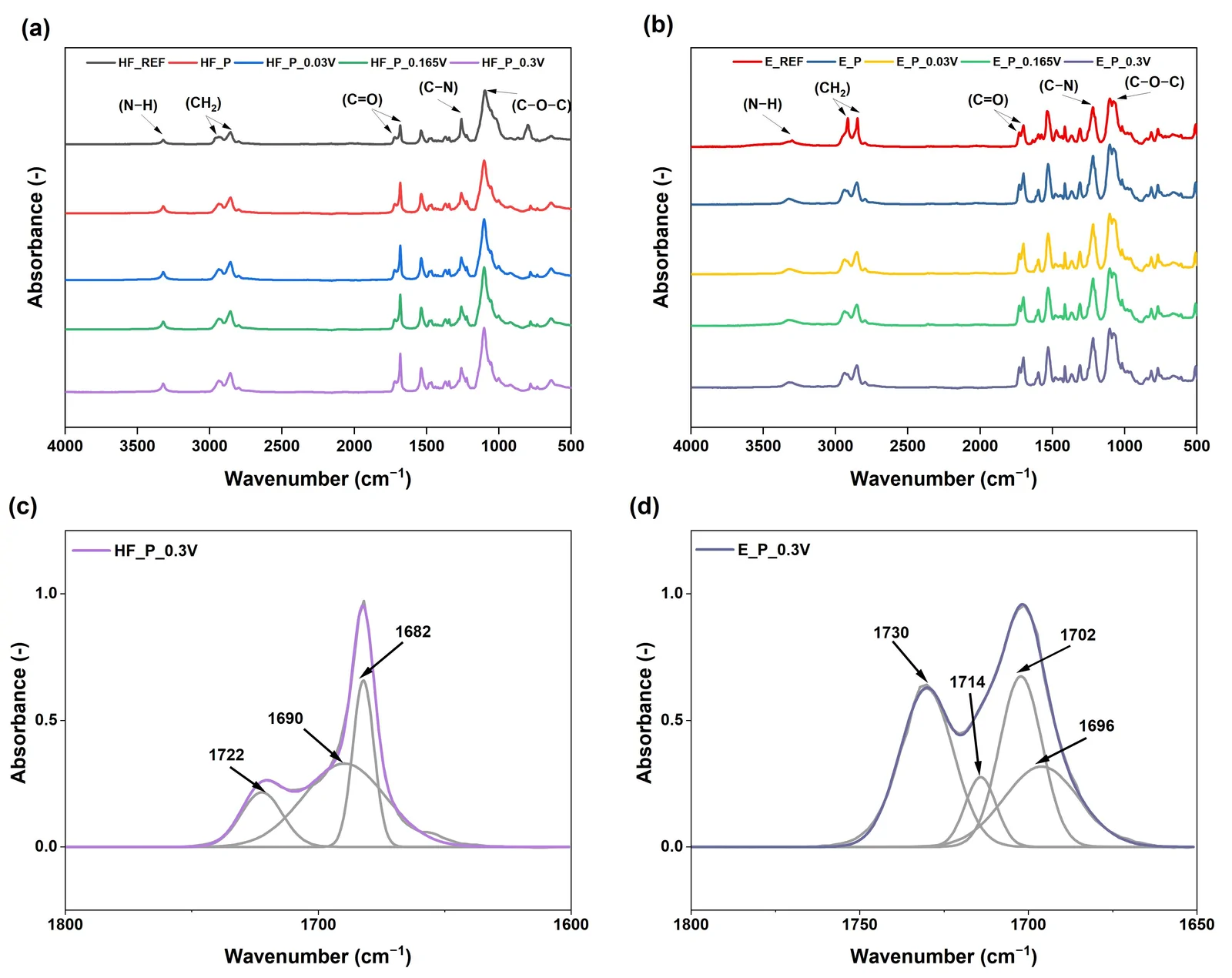
FTIR spectra recorded for HF (**a**) and E (**b**) TPU series, as well as representative peak deconvolution of the carbonyl band for HF (**c**) and E (**d**) TPU samples processed with the addition of 0.3 wt% vanillin.

The FTIR results, which are presented in Figure 3 and Table 2, confirm the presence of urethane linkages in both TPU systems: HF and E. In the case of the HF samples, the absence of the OH stretching band (~3600 cm^−1^) and the asymmetric NCO stretching band (2270 cm^−1^) further confirms that the two-step synthesis was carried out properly, with complete reaction between hydroxyl and isocyanate groups [29].

In the FTIR spectra of both series (Figure 3a,b), the following characteristic bands were identified: N–H stretching vibrations at ~3300 cm^−1^, asymmetric and symmetric CH_2_ stretching vibrations at 2860 and 2940 cm^−1^, the carbonyl (C=O) stretching band in the range of 1600–1800 cm^−1^, the C–N stretching band at ~1500 cm^−1^, and the ether (C–O–C) stretching vibration in the range of 1000–1100 cm^−1^. The presence of these bands in all samples—both unprocessed and processed, with and without vanillin as an additive stabilizer—confirms that processing did not lead to the formation of new bands or the disappearance of existing ones. No distinct vanillin-related bands were detected in the FTIR spectra of the processed samples, which is consistent with its low concentration (up to 0.3 wt%) relative to the TPU matrix. The main characteristic carbonyl band of vanillin, the aldehyde C=O stretch at ~1663 cm^−1^ and the phenolic C–O stretch at ~1261 cm^−1^ [30], would in any case overlap with the much more intense urethane C=O and C–N bands in these spectral regions.

The differences caused by processing and by the presence of the additive can only be detected through a detailed analysis of the carbonyl band, performed by deconvolution, as shown on Figure 3c,d and Figures S1 and S2.

It should be highlighted that the HF and E TPU systems differ in structural and compositional aspects beyond the aromatic/aliphatic character of the HS (including HS content), as well as soft-segment chemistry and processing conditions (see Section 2.3. and Figure 2). The two systems are therefore treated here as independent TPUs. Comparing the spectra of HF and E TPU series, it can be seen that the fingerprint region area, in the range of 500–1500 cm^−1^, is much more extensive in the case of the E TPU samples. This can be attributed, at least in part, to the aromatic character of the commercial Elastollan^®^ 1185 A, whereas the HF series based on an aliphatic diisocyanate mixture, exhibits a simpler pattern in this region. The complexity observed for the E series may be connected with the presence of the aromatic ring, as confirmed by, e.g., the C–H bending vibration in the range of 750–800 cm^−1^. Additionally, the C–O–C band, located in the 1000–1100 cm^−1^ region, exhibits a different shape between the two TPU series. In the HF series, it appears as a single, sharp peak, whereas in the E series it presents as two closely overlapping bands. It may be resolved into a component at ~1104 cm^−1^ assigned to the ether C–O stretching of the poly-ether soft segment, and a component at ~1077 cm^−1^ assigned to the urethane C–O stretching of HS [24].

The deconvolution of the carbonyl peak area lead to determine the relative proportions of hydrogen-bonded and unbonded C=O groups, which in turn allowed for the calculation of the carbonyl index (R), DPS, and DPM (values summarized in Table 2). Representative deconvolution results are shown in Figure 3c,d for the samples containing 0.3 wt% vanillin, while the spectra for all samples are provided in Figures S1 and S2. Across all samples, the HF series exhibited noticeably higher R (3.61–4.70) and DPS (0.783–0.824) values than the E series (R 1.96–2.31, DPS 0.663–0.698), which may be consistent with, among other factors, to the aliphatic, linear structure of the hard segments in the HF bio-TPU system. This trend is in agreement with previous FTIR studies, where a similar phenomenon was explained by the enhanced packing density of the aliphatic hard segments, leading to greater phase separation [31].

Melt-processing appeared to affect the HF and E TPU series in opposite ways, in the HF bio-TPU system, R and DPS showed a slight increase after processing, while in the E TPU series both parameters slightly decreased. This may be attributed to the different thermal histories of the reference materials—HF_REF was used directly after casting and curing, and melt-processing could lead to chain reorganization toward more phase-separated structure, similar to an annealing effect. In contrast, E_REF is a commercial pellet that had already undergone an optimized industrial thermal history.

A similar, though modest, divergence was observed in the case of increasing vanillin content, where R and DPS showed a slight increase for the HF bio-TPU series and a small decrease for the E TPU series. This may result from the aromatic character of vanillin; in the E TPU series, interactions between vanillin’s aromatic ring and the MDI aromatic ring could promote disrupted packing of hard domains, whereas in the aliphatic hard segments of the HF series, vanillin may not interfere with HS packing. Previous studies on vanillin-containing polyurethane systems have similarly demonstrated that the incorporation of vanillin-derived aromatic structures can modify, e.g., intermolecular interactions or hydrogen bonding, with the direction and magnitude of these changes strongly dependent on the polyurethane structure, as well as the form in which vanillin is introduced [21]. It should be noted, however, that these changes in DPS do not exceed 5% in either series, and may therefore have limited impact on the application-relevant performance of both bio-TPU and commercial-TPU systems.

**Figure 4:**
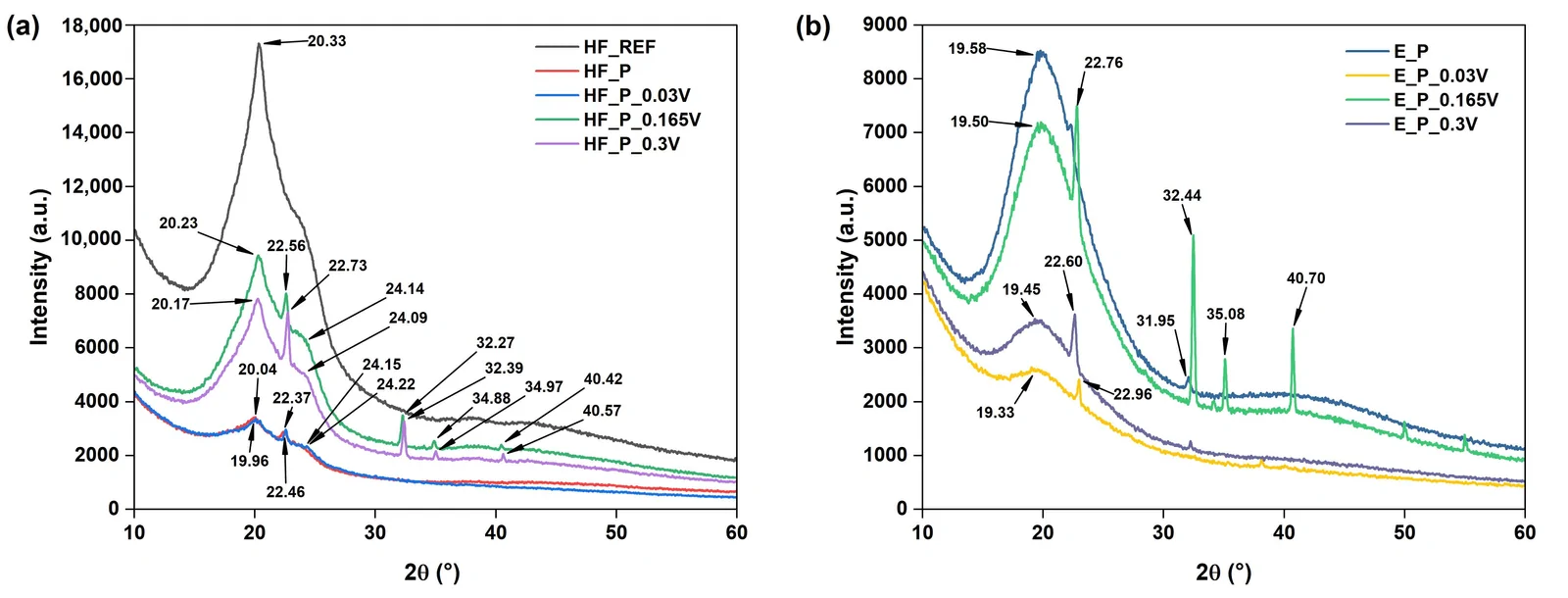
XRD patterns recorded for HF (**a**) and E (**b**) TPU series.

Thermoplastic polyurethane elastomers exhibit a segmented structure, which can be confirmed and analyzed using X-ray diffraction (XRD). In this work, XRD was used to determine the influence of both the effect of melt processing and additive incorporation on the morphology of TPUs. Results are presented in Figure 4 as XRD patterns. Processing and vanillin addition into the HF bio-TPUs series caused the changes in the morphological behavior of the materials. Analyzing reference material (HF_REF), the intense and relatively broad reflection with a maximum at 2θ value at ~20.33° and maximum at reflection shoulder ca. at 24° indicated a semicrystalline structure, but with the largest contribution of amorphous phase. The crystalline structure of polyurethanes might be attributed to both the soft and hard segments [25]. Two reflections at 2θ values of approximately 20° and 23° were also observed for HF TPUs samples after processing and contained vanillin (Figure 4a). For HF_P_0,165 and HF_ P_0.3 TPUs, additional minor reflections at approximately 23°, 32°, 34°, and 40° were registered. The XRD pattern of vanillin was presented in Figure S3 and revealed that the main reflection occurs at a 2θ value of 13.07°, and less intense signals at 23.8° and 39.8° and is consistent with the literature [32]. Notably, the XRD pattern of crystalline vanillin may vary depending on its crystalline form.

Analyzing Figure 4b, the highly amorphous structure of commercial E TPU series was revealed. As we mentioned in our previous work [25], the degree of crystallinity of polyurethane depends on the components used in their synthesis, which can explain the morphological differences between the synthesized HF bio-TPU series and the petrochemical based TPU. Vanillin addition was reflected in the appearance of diffraction signals at approximately 23°, 32°, 35°, and 41°, comparable to those observed for the HF bio-TPU series.

Since this study focuses on enhancing the thermo-oxidative stability of TPUs, it is also important to assess the thermal stability of the materials under inert conditions, as this provides a baseline for their behavior in the absence of oxidative degradation pathways. The TGA measurements presented in this section were performed under a nitrogen atmosphere and therefore primarily reflect thermal degradation processes. The oxidative stability of the materials is discussed in the OOT in Section 3.

**Figure 5:**
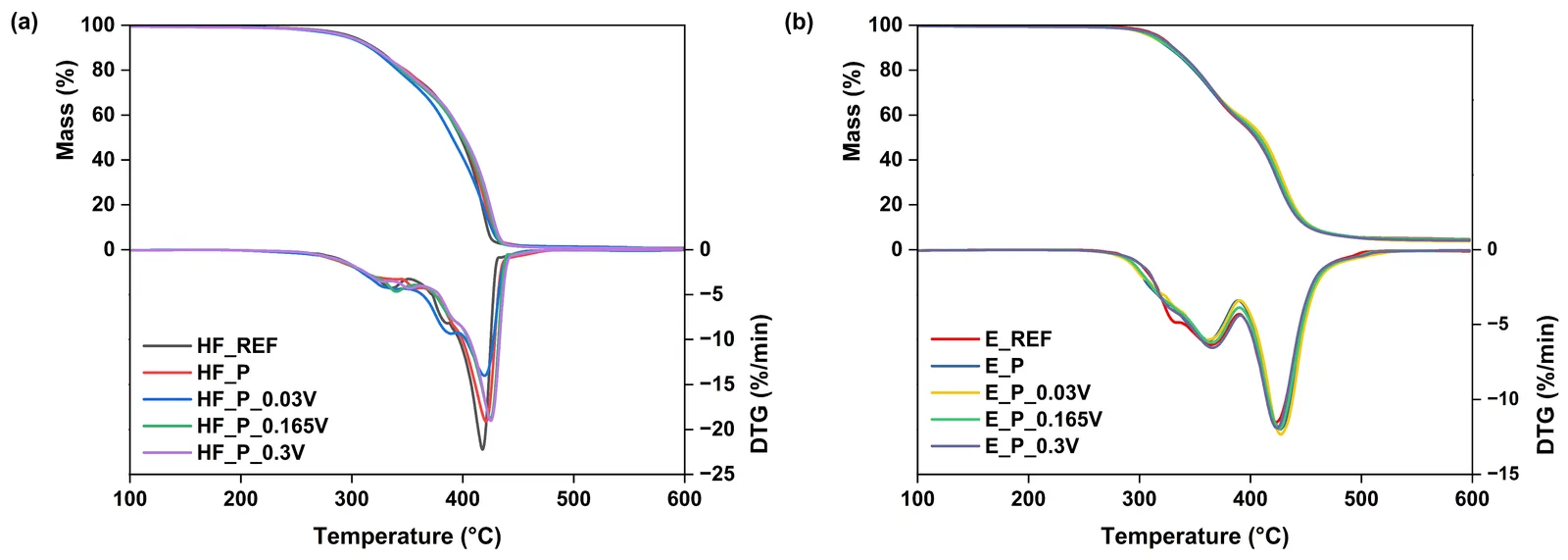
TG and DTG curves plotted for HF bio-TPU (**a**) and E TPU (**b**) series.

In the TGA results (Figure 5 and Table 3), a noticeable phenomenon can be observed—the commercial TPU system (E) exhibits higher thermal stability than the HF bio-TPU series (Figure 5), consistent with the general trends discussed in the FTIR section. This is broadly consistent with the typical behavior of aromatic-based systems—which tend to enhance thermal stability of the material [33], although other compositional differences between the two studied TPUs cannot be excluded. Comparing the T_5%_ parameters, the difference between HF_REF and E_REF is 18 °C, while the ash residue of the E series is 6–8 times higher than that of aliphatic-based HF system. A general decrease in ash residue was observed in both TPU series after processing without additives (HF: from 0.73 to 0.65%; E: from 4.62 to 4.22%). The overall low ash-residue values observed across the HF bio-TPU series (under 1%) are typical for aliphatic polyurethane systems, which generally exhibit a low char-forming tendency, in contrast, aromatic-based TPU systems typically yield higher ash residue due to the char-forming capacity of the aromatic ring [34]. In the HF series, the changes associated with vanillin content are minor (0.57–0.65%) and do not show a clear concentration-dependent trend. In the E series, the ash residue also varies without a clear trend upon vanillin addition and notably the maximum value is observed at 0.165 wt% vanillin (E_P_0.165V: 4.53%), which differs from the trend seen for T_5%_ and DPS, where the highest values were recorded for samples with 0.3 wt% vanillin (see FTIR section for a discussion of the magnitude of these changes). This is likely due to the higher sensitivity of ash residue to minor differences between individual samples than the other parameters.

DTG curves (Figure 5) show a two-step thermal degradation pattern, typical for TPU systems, where the first stage is connected with dissociation of urethane linkage (hard segment), and the second stage is related to degradation of the soft segment [35]. T_max1_ differs significantly between the bio-TPU and commercial TPU series, which can be consistent with urethane linkages formed from aromatic isocyanates generally exhibiting more thermally durable characteristics than those formed from aliphatic ones, although this effect may also be associated with other factors that distinguish the two TPU systems. The first degradation step (HS) also proceeds faster in the E series (DTG_1_: −6.0 to −6.5%/min) than in the HF series (−3.2 to −4.6%/min), despite occurring at a higher temperature. Tmax_2_, however, remains similar in both systems, indicating that the polyether-based soft domains degrade within a similar temperature range in both cases. In terms of rate, DTG_2_ is higher for the HF series (−14.0 to −22.1%/min) than for the E series (−11.5 to −12.3%/min), but notably the DTG_2_ values are nearly consistent across all E samples, whereas in HF they vary more markedly between samples. In both series, DTG_2_ proceeds at a higher rate than DTG_1_, which confirms that the SS degradation step is the dominant mass-loss event. This may also be partially attributed to the different HS contents of the two materials (~31 wt% for HF, ~40 wt% for E); therefore, the higher concentration of SS in the HF series leads to a more intense DTG peak for the SS-related degradation step, consistent with similar findings in the literature, where HDI- and MDI-based TPUs with different HS content were explored [33]. The overall differences in DTG peak temperatures, shapes, and rates, between the two bio-TPU and TPU series may reflect the underlying differences in HS/SS organization, as discussed in the FTIR section above.

**Figure 6:**
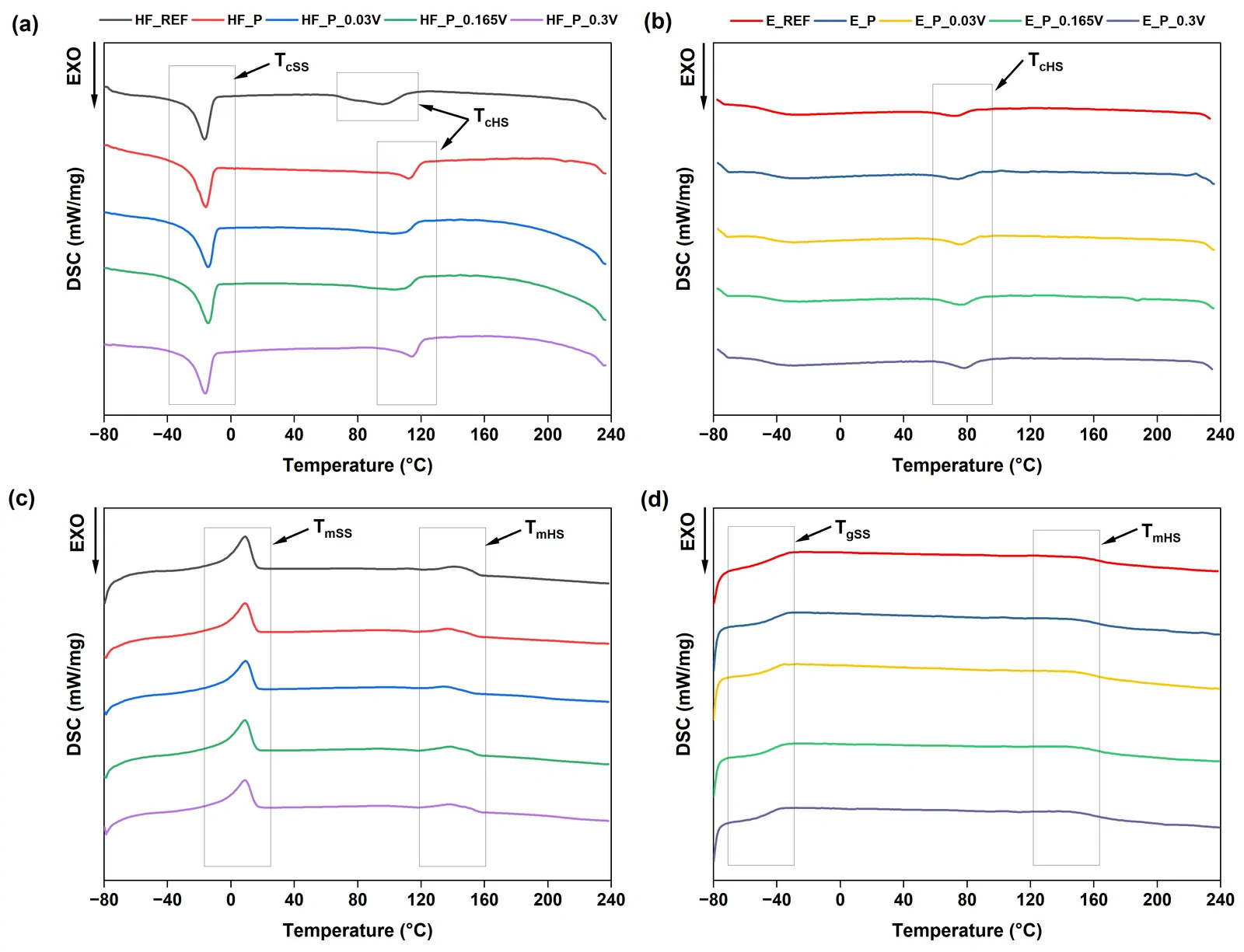
DSC curves of HF bio-TPU and E TPU series recorded during (**a**,**b**) cooling and (**c**,**d**) second heating cycles.

**Figure 7:**
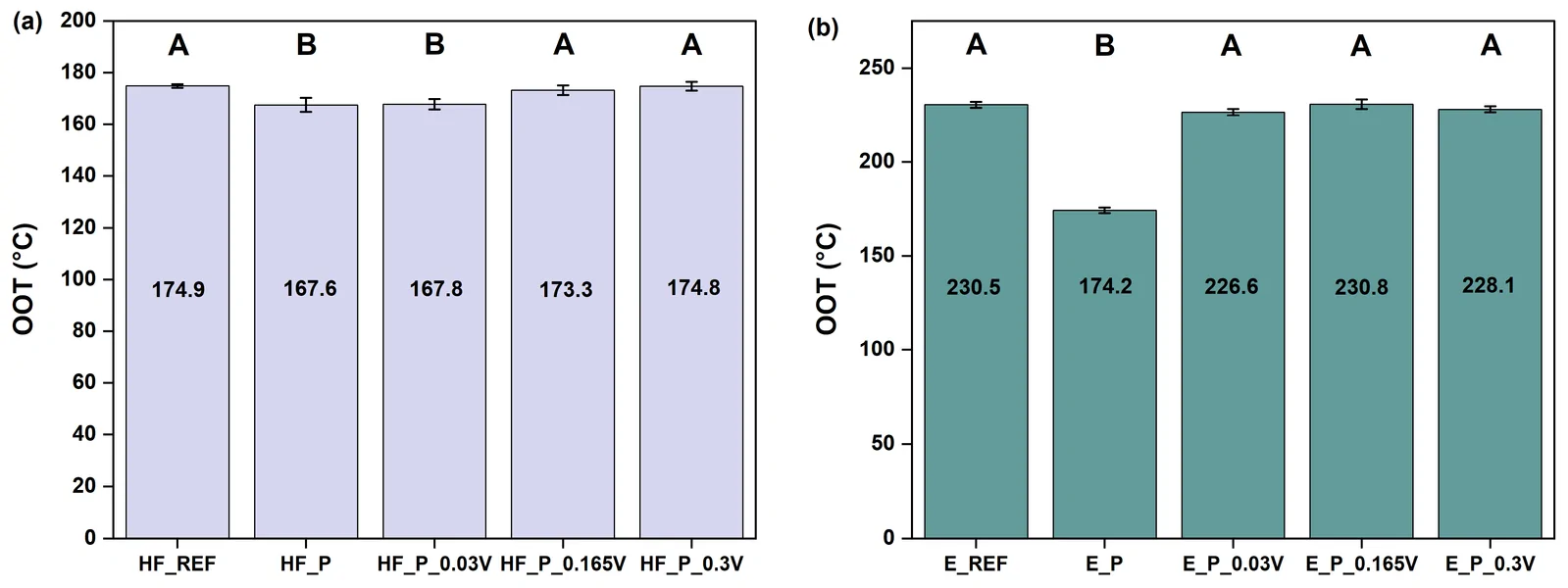
OOT results presented for the HF (**a**) and E (**b**) TPU series. Error bars represent standard deviation (*n* = 3). Different letters (A/B) above the bars indicate statistically significant differences within each series (one-way ANOVA followed by Tukey’s post hoc test, *p* < 0.05).

The incorporation of additives into polymers may affect their processing behavior and final properties, and can be indirectly assessed using differential scanning calorimetry (DSC). In this work, DSC was used to evaluate the effect of vanillin addition on the thermal behavior of TPUs during processing and on the overall thermal properties of the synthesized HF bio-TPU and commercial E TPU series, as well as to determine the oxidation onset temperature (OOT). The DSC thermograms are presented in Figure 6, while the determined glass-transition temperatures of the soft segments (T_gSS_), the melting/crystallization temperatures of the soft (T_mSS_/T_cSS_) and hard (T_mHS_/T_cHS_) segments, and melting/crystallization enthalpies of the soft (ΔH_mSS_/ΔH_cSS_) and hard (ΔH_mHS_/ΔH_cHS_) segments are shown in Table 4, along with the OOT results presented in Figure 7.

DSC analysis revealed the semicrystalline nature of all samples from the HF series, the HF bio-TPU, and highly amorphous from the commercial E TPU series. Analysis of the DSC results showed that the crystallization behavior of HF TPU series depended on the vanillin content and mainly affected the changes in the crystallization temperature and enthalpy of the hard segments (Figure 6a,b). The HF bio-TPU exhibited an increase in the crystallization temperature of hard segments (T_cHS_) with increasing vanillin content, from 95.8 °C (for the reference bio-TPU) to 139.3 °C (for HF_P_0.3V), which indicates a facilitated crystallization process. Vanillin, due to its chemical structure and aromatic ring, may enhance the phase separation and, to some extent, facilitate the crystallization of the HS. This assumption is broadly consistent with the small increase in DPS values presented in Table 2, although, as noted in the FTIR section, these changes are relatively small. Processing of the bio-TPUs without the antioxidant additive caused a decrease in crystallization enthalpy, ΔH_cHS_, suggesting a decrease in intramolecular interaction. The effect of processing on the decrease in the melting and crystallization temperatures and enthalpies of the hard segments of the bio-TPU series (HF_REF and HF_P) were also observed in our previous work [25]. The decrease in melting enthalpy might be related to the decrease in the degree of crystallinity [36]. Addition of vanillin at concentrations up to 0.165 wt% caused an increase in ΔH_cHS_ from 4.9 J/g (HF_P) to 10.6 J/g, which can be the effect of the facilitated crystallization of hard segments. Analysis of the effect of vanillin addition on the crystallization behavior of the soft segments for series HF bio-TPU showed no significant influence on either the crystallization temperature or enthalpy.

Analysis of the exothermic curves of the petrochemical E TPU series (Figure 6b) revealed the amorphous soft phase and partially crystalline hard phase. Application of vanillin as a processing stabilizer—antioxidant—caused an increase in the crystallization temperature of the hard segments (T_cHS_) from 72.0 °C (E_REF) to 79.0 °C (E_P_0.03V), and in crystallization enthalpy of hard segments, from 3.8 J/g (E_REF) to 6.5 J/g (E_P_0.165V). Notably, according to the literature, the crystallization of vanillin occurs approximately at 84 °C [37], a higher temperature than in the case of hard segments in petrochemical TPU. It suggests that vanillin may acts as a nucleation agent. An increase in mentioned parameters was also observed when considering the influence of the processing on thermal behavior of TPU, which has not been observed for the HF bio-TPU series. This phenomenon indicates an improvement in the structural ordering, as well as the presence of hard segment crystallites similar in size and shape.

Considering the endothermic curves of the HF bio-TPU series (Figure 6c), melting transitions corresponding to both the soft and hard segments were observed despite the addition of vanillin as a stabilizer, whereas the commercial TPU series (Figure 6d) exhibited only a melting transition associated with the hard segments. Analysis of the thermal transitions of the HF series showed that the melting temperature of the soft segments was approximately 9 °C, regardless of the vanillin addition, while the melting enthalpy ranged from 19.5 to 23.2 J/g, indicating slight changes in the arrangement of the soft segment chains. The melting temperature of the hard segments in the bio-TPU series decreased with increasing vanillin content, from 143.2 to 139.3 °C, while no consistent trend was observed in the melting enthalpy (ΔH_mHS_). A decrease in the melting temperature of the hard segments with maintained enthalpy may be caused by the formation of smaller crystalline forms. The commercial TPU containing vanillin exhibited a lower melting temperature of HS accompanied by an increase in melting enthalpy (ΔH_mHS_) from 2.0 J/g to 3.2 J/g (Table 4), which can be explained by a higher crystallinity in the hard domain, combined with a smaller size of the crystalline structures [38] in comparison to the E_REF. The amorphous behavior of the soft segments in commercial TPU was reflected by glass transition temperatures ranging from −46.3 to −41.6 °C. T_gSS_ significantly decreased due to processing.

As mentioned in the introduction, melt processing of polymers is typically performed at elevated temperatures under shear, and thermo-mechanical and thermo-oxidation processes may therefore occur simultaneously. Both processes can be induced by free radicals formed due to heat, trace impurities, and other agents used during the synthesis. Overall, mechanical stress may favor degradation by breaking the polymer chains and generating radicals via a mechanochemical mechanism. These radicals may readily react with oxygen, generating peroxyl radicals (ROO**^•^**) and consequently lead to hydrogen abstraction from the polymer chains. caused the hydroperoxides formation, which may quickly decompose to form alkoxy (RO**^•^**) and hydroxy (**^•^**OH) radicals [4]. Considering poly(ether-urethane) materials, thermo-oxidation may occur in both soft and hard segments [39,40].

The influence of vanillin as a natural antioxidant in the melt processing of TPUs was assessed based on the oxidation onset temperature (OOT) as presented in Figure 7. At first glance, processing of both series of TPU caused a decrease in OOT, indicating that oxidation occurred at a lower temperature. Considering petrochemical TPU (E series), OOT decreased by approximately 55 °C. The addition of vanillin increased OOT to a value comparable to that of the reference material E_REF and confirmed its antioxidant activity. Thereby vanillin can be classified as a mildly sterically hindered phenol (Ar−OH), which acts by efficiently scavenging peroxyl radicals (ROO**^•^**), thereby preventing them from abstracting hydrogen atoms from the polyurethane chains and forming the stable phenoxy radicals (Ar−O**^•^**). The resulting phenoxyl radical, Ar−O**^•^**, is resonance-stabilized and therefore much less likely to propagate the radical chain reaction. By scavenging radicals involved in the initiation and propagation steps of oxidation, phenolic –OH groups suppress autoxidative chain reactions [41,42]. Experimentally, this enhanced oxidative stability of both TPU series, after vanillin addition, was reflected by an increase in the OOT, since a higher temperature is required to initiate rapid oxidative degradation. However, as shown by the statistical analysis below, this recovery followed a concentration-dependent pattern rather than a continuous increase, differing between the two TPU series. In contrast, the aldehyde group derived from the vanillin structure is not generally considered a radical scavenger. However, the aldehyde group can undergo oxidation to the corresponding carboxylic acid, meaning that it may participate in oxidation reactions through a mechanism distinct from the phenolic antioxidant pathway.

One-way ANOVA followed by Tukey’s post hoc test was performed on all TPU samples in each series to assess the statistical significance of differences in OOT values. In the HF series, ANOVA revealed statistically significant differences among the samples (F(4, 10) = 11.41, *p* = 0.0011). Tukey’s test grouped HF_REF, HF_P_0.165V, and HF_P_0.3V together, with significantly higher OOT values than samples HF_P and HF_P_0.03V, that did not differ greatly from each other. This confirms that, in the HF bio-TPU series, a vanillin concentration of at least 0.15 wt% was required to restore OOT to a level comparable with the unprocessed reference. ANOVA also detected statistically significant differences among the samples in the E TPU series (F(4, 10) = 548.71, *p* < 0.00001). Tukey’s test showed that E_P was notably lower than all the other samples. Meanwhile, E_REF, E_P_0.03V, E_P_0.165V, E_P_0.3V did not differ markedly from each other. This indicates that, in contrast to the HF series, even the lowest tested vanillin concentration (0.03 wt%) was sufficient to restore OOT to a level statistically similar to that of the unprocessed reference material in the E TPU series.

In general, the HF bio-TPU series exhibited significantly lower OOT than the petrochemical TPU. This phenomenon may be related, among other factors, to differences in the chemical structure of the soft and hard segments of each TPU series. The thermal stability of aromatic-based polyurethanes (E TPU series) with the soft segments built from poly(tetramethylene ether) glycol (PTMG) residue polyether is higher compared to the aliphatic-based polyurethanes (HF bio-TPU series) with soft segments consisting of poly(trimethylene ether) glycol (PO3G) due to the greater chain length. On the one hand, the aromatic ring can enhance the thermal stability of the TPU. It is known that the oxidation of the soft segments plays a major role in the aging of polyether-based TPUs [43] and a decrease in the monomeric unit length of the diol causes a deterioration in the thermo-oxidative stability of TPU. For comparison, the literature [7] reports an OOT of 254 °C for a polycaprolactone-based TPU (Pearlthane^®^ 11H94). Vanillin at concentrations of 0.165 wt% and 0.3 wt% allowed satisfactory oxidative stability to be maintained after processing. According to the literature it is known that the kinetics of different antioxidant may be completely different [7]. It should be highlighted that, although the radical scavenging and chain-related mechanisms discussed above are consistent with the OOT results, they were not confirmed experimentally in this study. Therefore, they are presented as proposed explanations.

**Figure 8:**
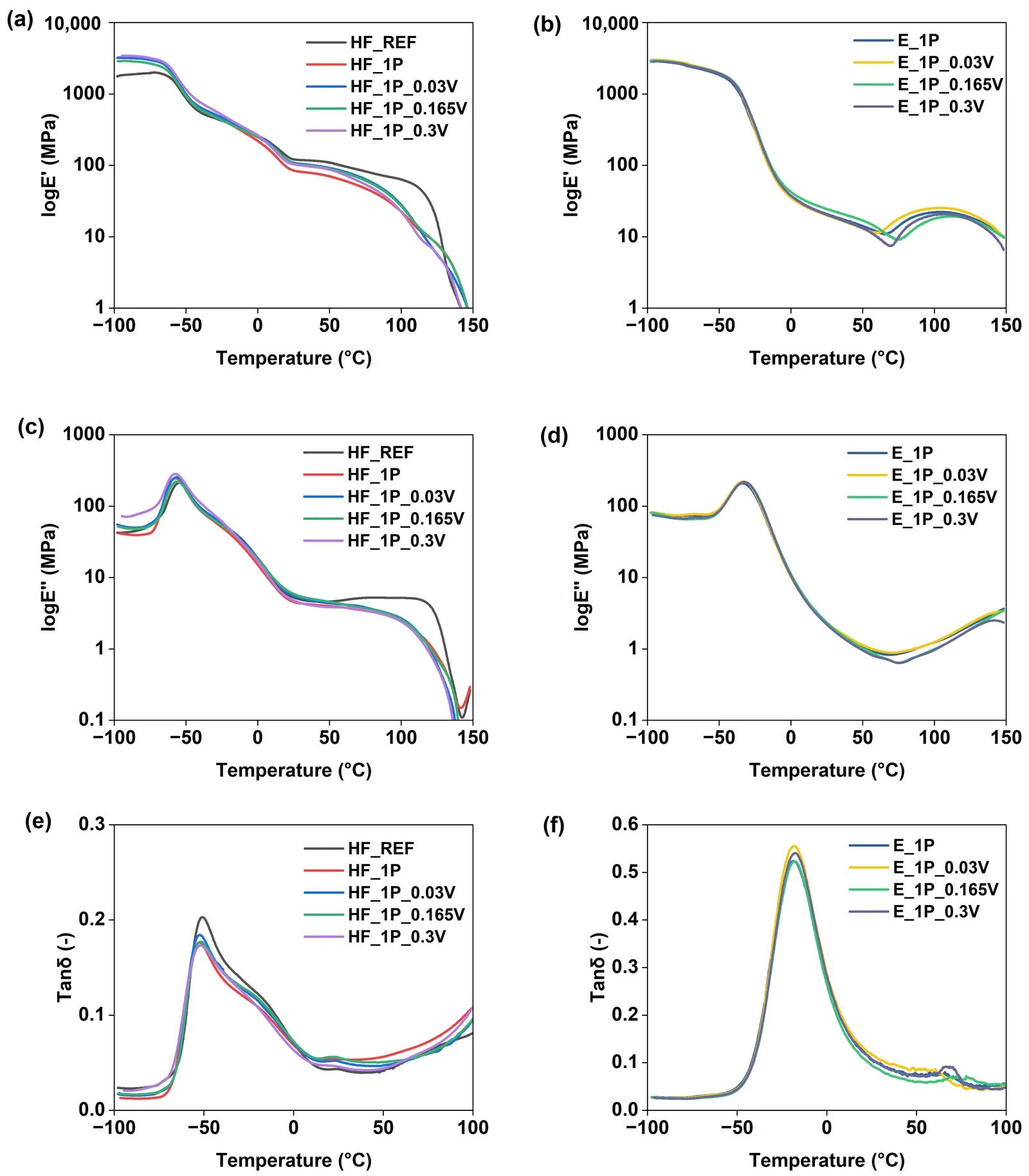
DMTA curves of the HF and E series showing log E′ (**a**,**b**), log E″ (**c**,**d**), and tan δ (**e**,**f**) as a function of temperature.

Dynamic mechanical thermal analysis (DMTA) provides valuable insight into the temperature-dependent viscoelastic behavior of TPUs. As illustrated in Figure 8, both TPU series demonstrate a pronounced decrease in storage modulus (E’) with increasing temperature, accompanied by maxima in the loss modulus (E’’) and damping factor (tanδ), which are linked to relaxation processes and the dissipation of mechanical energy.

When comparing the bio-TPU and commercial TPU series, the most noticeable difference lies in the values of the glass transition temperature of soft segments (T_gSS_), which for HF series ranges from −52.6 to −50.8 °C, whereas for the E series it occurs in the range of −19.7 to −17.6 °C. This phenomenon is consistent with the general relationship between the loss-peak temperature and the degree of phase separation in PUs, where a higher peak temperature reflects a lower degree of phase separation [44]. These results are broadly consistent with the DPS values obtained from FTIR analysis, where the E series exhibited a lower DPS than the HF bio-TPU series, suggesting that a larger proportion of HS mixed within the SS matrix affects chain mobility, thereby promoting higher T_gSS_ values. Similarly, the maximum tanδ value (Figure 8e,f) is also substantially higher for the E TPU series (0.522–0.555) than for the HF bio-TPU series (0.173–0.203), indicating much more pronounced mechanical damping in the glass-transition region. Together with the higher T_gSS_ and the FTIR-derived DPS values, this behavior supports a greater extent of hard and soft segment mixing in the commercial TPU. The considerably higher damping-factor values of the E TPU series are also application-relevant, as such materials exhibit better damping properties, which can determine their potential industrial applications. At room temperature, the HF bio-TPUs series exhibit markedly higher E’ values (85–122 MPa) than the E sample series (20–25 MPa), demonstrating the higher room-temperature stiffness of the HF series. Since both materials are well above their T_gSS_ values at 25 °C, these differences cannot be attributed to T_gSS_ and are most likely also due to differences in HS chemical structure and morphological organization.

Melt-processing induced changes in the viscoelastic properties of the HF bio-TPU series. The T_gSS_ slightly decreased (from −50.8 to −52.5 °C), which is consistent with the DPS trend and may indicate improved phase separation. A similar decrease in T_gSS_ has been reported for TPU composites after annealing [45]. The maximum of the tanδ peak also decreased (from 0.203 to 0.176), likewise indicating lower energy dissipation in the glass transition region, consistent with a better separated microphase morphology of the bio-TPU. These changes are also visible in the case of E’ and E’’ moduli. The storage modulus in the glassy state (E’_max_) increased markedly from 2010 to 3194 MPa (Figure 8a,b), which indicates a higher low-temperature stiffness of the material in this temperature range, although at room temperature, E’ exhibited a decrease from 122 to 85 MPa (Figure 8c,d). As mentioned in Section 2, the E TPU series was supplied in pellet form, and the DMTA test was therefore not possible prior to processing. Consequently, the DMTA results for this series begin with E_P.

Regarding the effect of vanillin addition on the DMTA results (Table 5), the T_gSS_ in the HF series remained stable across all vanillin concentrations (from −52.6 to −51.8 °C) without a clear concentration-dependent trend. Importantly, the addition of vanillin partially restored E’_25 °C_ to 104–109 MPa compared with 85 MPa for HF_P, although the values remained below those of HF_REF (122 MPa). This partial recovery is consistent with the stabilizing effect observed in the OOT results, particularly at higher vanillin concentrations (0.165 and 0.3 wt%). In contrast, the E series exhibited a small but distinct increase in T_gSS_ with vanillin concentration, from −19.7 °C (E_P) to −17.6 °C (E_P_0.3V). Additionally, the tanδ peak maximum does not show a monotonic trend, increasing at the 0.03 wt% vanillin concentration (0.555), decreasing for the sample with 0.165 wt% of vanillin (0.523), and partially recovering at 0.3 wt% (0.541). Similarly, only minor changes in E’’ were observed (209–222 MPa), indicating that vanillin has no systematic effect on the maximum viscous response.

In general, the DMTA results provide additional evidence of the differences in phase separation between the two analyzed series—HF and E—which were detected previously by FTIR spectroscopy. The most significant difference lies in the T_gSS_ values (with a ~30 °C difference between the HF and the E), as well as in the maximum of the tanδ peak (more than twice as high for the E series), which supports the conclusion of more pronounced phase mixing in the commercial TPU, as established through carbonyl peak deconvolution in the FTIR section.

It is obvious that additives incorporated into the polymer matrix may result in changes in physical properties. It depends on the main function of the additive, its chemical structure and amount. In general, tensile properties of polyurethane materials depend on many factors, e.g., chemical structure of the polymer, morphology, molecular weight or hard and soft segments content, molar ratio of isocyanate to hydroxyl groups as well as synthesis and processing methods [46].

In this work, the effect of antioxidant on the tensile properties (tensile strength, TS_b_, and nominal elongation at break, ε_b_) and hardness (H) was also investigated. Results, together with the corresponding statistical analysis (one-way ANOVA followed by Tukey’s post hoc test), are presented in Table 6. At first glance, the vanillin addition caused a slight decrease in tensile properties of the HF bio-TPU series, especially considering the elongation at break. This observation suggests a mainly physical interaction between vanillin and bio-TPUs. A similar trend was observed in the case of E TPU series, where the addition of vanillin caused a slight decrease in tensile strength and elongation at break.

To assess the statistical significance of these trends, one-way ANOVA followed by Tukey’s post hoc test was performed separately for each series and parameter. Significant differences were found for TS_b_ and ε_b_ in both series (for HF: TS_b_: F(4, 20) = 12.76, *p* < 0.0001; ε_b_: F(4, 20) = 19.56, *p* < 0.0001; for E: TS_b_: F(3, 16) = 7.11, *p* = 0.00298; ε_b_: F(3, 16) = 6, 38, *p* = 0.00475). In the HF bio-TPU series, vanillin-containing samples showed TS_b_ statistically comparable to HF_REF, while ε_b_ remained intermediate between HF_REF and HF_P. In the E series, both TS_b_ and ε_b_ decreased significantly at the highest vanillin concentration (0.3 wt%) relative to E_P and E_P_0.03V.

Considering the HF bio-TPU series, slight changes may be observed analyzing the reference (HF_REF) material and processed bio-TPUs (HF_P). Analyzing elongation at the break of the HF bio-TPU series, it can be seen that this parameter did not change regardless of the vanillin content. Considering the E TPU series, with increasing vanillin content a decrease in TS_b_ (24.84–20.93 MPa) and ε_b_ (1425–1247%) were noticed, which may be related to a more poorly separated microphase morphology. Under melt-processing conditions such as high temperature and shear stress, radical-mediated reactions may promote degradation, as well as branching or crosslinking of the polymer chain. In this case, the most probable scenario is related with the chain scission occurring during the processing of the E TPU series, thereby leading to the reduced tendency toward microphase separation. These results are again consistent with the DPS values obtained from FTIR analysis and DSC results.

As we mentioned in our previous work [47], hardness can be defined as a stress which is needed to plastically deform the material and is closely related to the morphological structure and tendency to microphase separation. Considering hardness, one-way ANOVA revealed no statistically significant differences among the samples in either series (HF: F(4, 45) = 1.35 *p* = 0.265; E: F(3, 36) = 2.58, *p* = 0.068), indicating that vanillin addition did not significantly affect the hardness of the analyzed bio-TPU and TPU series. HF bio-TPU materials exhibited hardness ranging from 86.9 to 89.9 ShA, while E TPUs series from 76.8 to 80.6 ShA. A slight decrease in hardness of the E TPU series may be associated with weaker intermolecular cohesive forces between the polymer chains and more poorly separated microphase morphology. Nevertheless, the incorporation of the vanillin did not result in significant changes in the mechanical properties of both series of TPU. Vanillin successfully fulfilled its primary function as a melt-processing stabilizer, hampering the rate of free-radical chain reactions in both series of TPUs.

## 4. Conclusions

Overall, the present study provided new insights into the incorporation of natural compounds as stabilizers in thermoplastic polymer systems. In this study, vanillin at three concentrations (0.03, 0.165, and 0.3 wt%) was used as a primary antioxidant for the thermo-oxidative stabilization of bio-TPU and commercial TPU systems during melt processing. Based on the presented results, we confirmed our hypothesis that vanillin, as a bio-based phenolic aldehyde, can be used as a natural antioxidant.

The FTIR results confirmed the chemical structure of the synthesized HF bio-TPU series and the commercial TPU, while simultaneously revealing the influence of vanillin on the degree of phase separation. It was found that vanillin addition enhanced phase separation in the HF TPU series. Both analyzed material series exhibited a segmented structure, which was confirmed by the TGA, DSC, and X-ray diffraction results. Thermal stability results for both series indicated that HF bio-TPU exhibited a lower onset temperature of thermal degradation (T_5%_), by approximately 18 °C, compared to the commercial TPU, which may be related, among other factors, to the aromatic character of its hard segments. The nature of aliphatic hard segments in HF bio-TPU led to a decrease in the melting temperature of the hard segments. Based on the DSC results we found that vanillin addition enhanced the crystallization process of the hard segments, as indicated by an increase in crystallization temperature. In general, DSC and XRD results revealed the semicrystalline structure of the HF_TPU series and almost amorphous nature of the E_TPU series. Nevertheless, the HF bio-TPU series was characterized by the presence of an ordered phase in soft and hard segments. Regardless of the TPU series, melt processing caused a decrease in the oxidation onset temperature (OOT), more pronounced for commercial TPU (OOT decreased by ca. 55 °C compared with the reference sample). The addition of vanillin in amounts above 0.03 wt% increased the OOT. These results confirmed that vanillin acts as a thermo-oxidative stabilizer during the melt processing of TPUs. DMTA results are consistent with OOT results and confirm a stabilizing effect of vanillin as a melt-processing stabilizer. The vanillin addition caused slight decreasing in the tensile properties, which suggests a mainly physical interaction between vanillin and TPUs. A modest decrease in tensile strength and elongation at break confirmed the importance of thermal stabilization of the material during processing to preserve the mechanical properties of the TPUs.

To conclude, the results confirmed that vanillin may be used as a melt-processing stabilizer and may protect TPU against thermo-oxidation. Nevertheless, the antioxidant activity of vanillin may be further enhanced by the chemical modification of an aldehyde group. Future work will focus on analyzing the impact of the modified bio-based stabilizer on processing and mechanical properties of TPUs, including their behavior under multicycle processing conditions.

## Figures and Tables

**Table 1:** Coding system and description of synthesized bio-TPU and commercial TPU.

Sample Code	Material	Processing/Additive
HF_REF	Synthesized bio-TPU based on 80%HDI/20% FLO	Unprocessed reference
HF_P		Processed without stabilizer
HF_P_0.03V		Processed with 0.03 wt% of vanillin
HF_P_0.165V		Processed with 0.165 wt% of vanillin
HF_P_0.3V		Processed with 0.3 wt% of vanillin
E_REF	Commercial TPU Elastollan^®^ 1185 A	Unprocessed reference
E_P		Processed without stabilizer
E_P_0.03V		Processed with 0.03 wt% of vanillin
E_P_0.165V		Processed with 0.165 wt% of vanillin
E_P_0.3V		Processed with 0.3 wt% of vanillin

**Table 2:** Comparison of R, DPS and DPM, calculated from carbonyl peak deconvolution of HF bio-TPU and E TPU series.

Sample Code	R	DPS	DPM
HF_REF	3.61	0.783	0.217
HF_P	4.24	0.809	0.191
HF_P_0.03V	4.45	0.816	0.184
HF_P_0.165V	4.51	0.819	0.181
HF_P_0.3V	4.70	0.824	0.176
E_REF	2.31	0.698	0.302
E_P	2.17	0.684	0.316
E_P_0.03V	2.04	0.672	0.328
E_P_0.165V	1.96	0.663	0.337
E_P_0.3V	2.03	0.669	0.331

**Table 3:** Thermal characteristics of HF and E TPUs based on TGA results.

Sample Code	T_2%_ (°C)	T_5%_ (°C)	T_max1_ (°C)	DTG_1_ (%/min)	T_max2_ (°C)	DTG_2_ (%/min)	Ash Residue (%)
HF_REF	266	300	335	−4.2	417	−22.1	0.73
HF_P	262	295	330	−3.2	420	−19.0	0.65
HF_P_0.03V	255	294	338	−4.4	420	−14.0	0.57
HF_P_0.165V	261	296	339	−4.6	424	−18.7	0.62
HF_P_0.3V	261	297	327/350	−3.5/−4.3	425	−19.0	0.58
E_REF	301	318	363	−6.3	423	−11.5	4.62
E_P	294	311	361	−6.1	427	−12.0	4.22
E_P_0.03V	293	311	360	−6.0	426	−12.3	3.74
E_P_0.165V	295	312	362	−6.2	425	−11.9	4.53
E_P_0.3V	296	317	364	−6.5	424	−11.9	4.09

T_2%_ and T_5%_ represent the temperatures corresponding to 2% and 5% mass loss, respectively. T_max1_ and T_max2_ refer to the temperatures of the first and second maximum peaks on the DTG curves, respectively, while DTG_1_ and DTG_2_ represent the corresponding maximum mass-loss rates.

**Table 4:** Thermal properties of HF and E TPUs based on DSC results.

Sample Code	T_cSS_ (°C)	ΔH_cSS_ (J/g)	T_cHS_ (°C)	ΔH_cHS_ (J/g)	T_gSS_ (°C)	T_mSS_ (°C)	ΔH_mSS_ (J/g)	T_mHS_ (°C)	ΔH_mHS_ (J/g)
HF_REF	−16.7	17.6	95.8	12.2	-	9.0	21.7	143.2	6.4
HF_P	−15.9	16.3	112.6	4.9	-	9.0	20.5	137.9	5.7
HF_P_0.03V	−14.3	17.9	108.4	6.0	-	9.1	23.2	135.8	4.1
HF_P_0.165V	−15.8	19.0	114.7	10.6	-	8.8	22.9	138.3	7.0
HF_P_0.3V	−16.1	17.4	114.4	8.6	-	8.9	19.5	139.3	6.2
E_REF	-	-	72.0	3.8	−43.7	-	-	155.5	0.6
E_P	-	-	74.6	5.8	−46.3	-	-	148.0	2.0
E_P_0.03V	-	-	79.0	6.0	−41.6	-	-	147.5	2.4
E_P_0.165V	-	-	75.8	6.5	−43.6	-	-	147.3	2.8
E_P_0.3V	-	-	78.1	6.4	−44.7	-	-	144.8	3.2

**Table 5:** Dynamic mechanical properties of HF and E based on DMTA results.

Sample Code	E’max (MPa)	E’_25 °C_ (MPa)	E’’max (MPa)	Tanδ (-)	T_gSS_ (°C)
HF_REF	2010	122	214	0.203	−50.8
HF_P	3194	85	248	0.176	−52.5
HF_P_0.03V	3257	109	254	0.185	−52.6
HF_P_0.165V	2913	106	221	0.177	−51.9
HF_P_0.3V	3451	104	283	0.173	−51.8
E_P	2883	21	209	0.522	−19.7
E_P_0.03V	3023	20	222	0.555	−18.4
E_P_0.165V	2953	25	217	0.523	−18.1
E_P_0.3V	2868	20	218	0.541	−17.6

**Table 6:** Mechanical characterization of HF and E TPUs based on tensile test results and hardness. Letters (A, B, C) indicate statistically significant differences within each series and parameter (Tukey’s test, *p* < 0.05).

Sample Code	TS_b_ (MPa)	ε_b_ (%)	H (ShA)
HF_REF	4.25 ± 0.21 A	36 ± 5 A	87.8 ± 4.6 A
HF_P	3.43 ± 0.21 B	18 ± 2 B	86.9 ± 3.0 A
HF_P_0.03V	3.99 ± 0.20 A	23 ± 2 B,C	88.7 ± 3.2 A
HF_P_0.165V	3.93 ± 0.16 A	23 ± 2 B,C	87.6 ± 2.8 A
HF_P_0.3V	4.08 ± 0.19 A	25 ± 3 C	89.9 ± 1.0 A
E_P	24.84 ± 0.93 A	1425 ± 57 A	80.6 ± 2.2 A
E_P_0.03V	24.16 ± 1.27 A	1418 ± 71 A	76.8 ± 4.3 A
E_P_0.165V	22.43 ± 1.12 A,B	1358 ± 53 A,B	78.5 ± 3.0 A
E_P_0.3V	20.93 ± 2.23 B	1247 ± 104 B	78.2 ± 2.2 A

## Data Availability

Data will be available on request.

## Supplementary Materials

The following supporting information can be downloaded at: https://www.mdpi.com/article/10.3390/polym18202539/s1, Figure S1: Deconvolution of the carbonyl band for HF bio-TPU series for (a) HF_REF, (b) HF_P, (c) HF_P_0.03V, (d) HF_P_0.165V, (e) HF_P_0.3V samples; Figure S2: Deconvolution of the carbonyl band for E commercial TPU series for (a) E_REF, (b) E_P, (c) E_P_0.03V, (d) E_P_0.165V, (e) E_P_0.3V samples; Figure S3: XRD pattern of vanillin; Table S1: Photographs of the bio-TPUs and TPUs presented in the paper; Table S2: Peak-fitting parameters (peak positions and maximum heights) and coefficient of determination (R2) for the Gaussian components obtained from deconvolution of the carbonyl band (1600–1800 cm^−1^), for HF and E TPU series; Table S3: The mean values of onset oxidation temperature (OOT) with standard deviations (*n* = 3) and results of Tukey’s post hoc test for the HF and E TPU series. One-way ANOVA: HF series, F (4, 10) = 11.41, *p* = 0.0011. E series, F (4, 10) = 548.71, *p* < 0.0001. Samples sharing the same letter within a series do not significantly differ according to Tukey’s post hoc test (*p* < 0.05).
