# Supplementary material for: Sustainable Approach to Thermoplastic Polyurethanes Stabilization: Vanillin as a Natural Additive in Melt-Processing
Source: Polymers (Basel). 2026 Oct 16;18(20):2539. doi: 10.3390/polym18202539 (PMC13709224; doi:10.3390/polym18202539)
Supplement: Supplementary file 1 [file polymers-18-02539-s001.zip › polymers-4569814-supplementary.pdf]

Supporting Information

Sustainable Approach to Thermoplastic Polyurethanes Stabilization: Vanillin as a Natural Additive in Melt-Processing

Joanna Smorawska-Kliza[1], Anna Jażdżewska[1] and Ewa Głowińska[1,*]

[1]Department of Polymer Technology, Faculty of Chemistry, Gdańsk University of Technology, 11/12 Gabriela Narutowicza Street, 80-233 Gdańsk, Poland

*corresponding author e-mail address: ewa.glowinska@pg.edu.pl

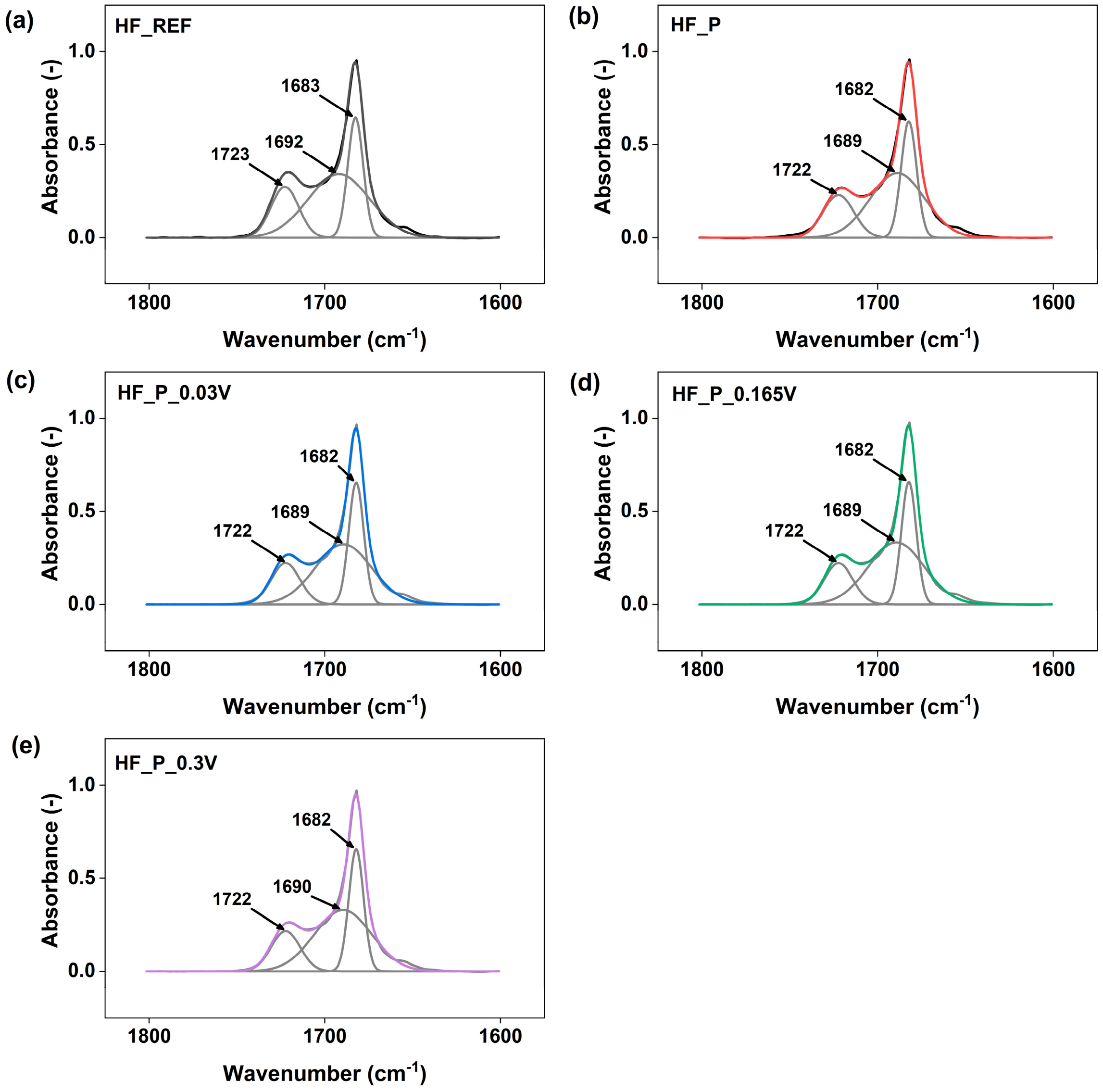

Figure S1. Deconvolution of the carbonyl band for HF bio-TPU series for **(a)** HF_REF, **(b)** HF_P, **(c)** HF_P_0.03V, **(d)** HF_P_0.165V, **(e)** HF_P_0.3V samples.

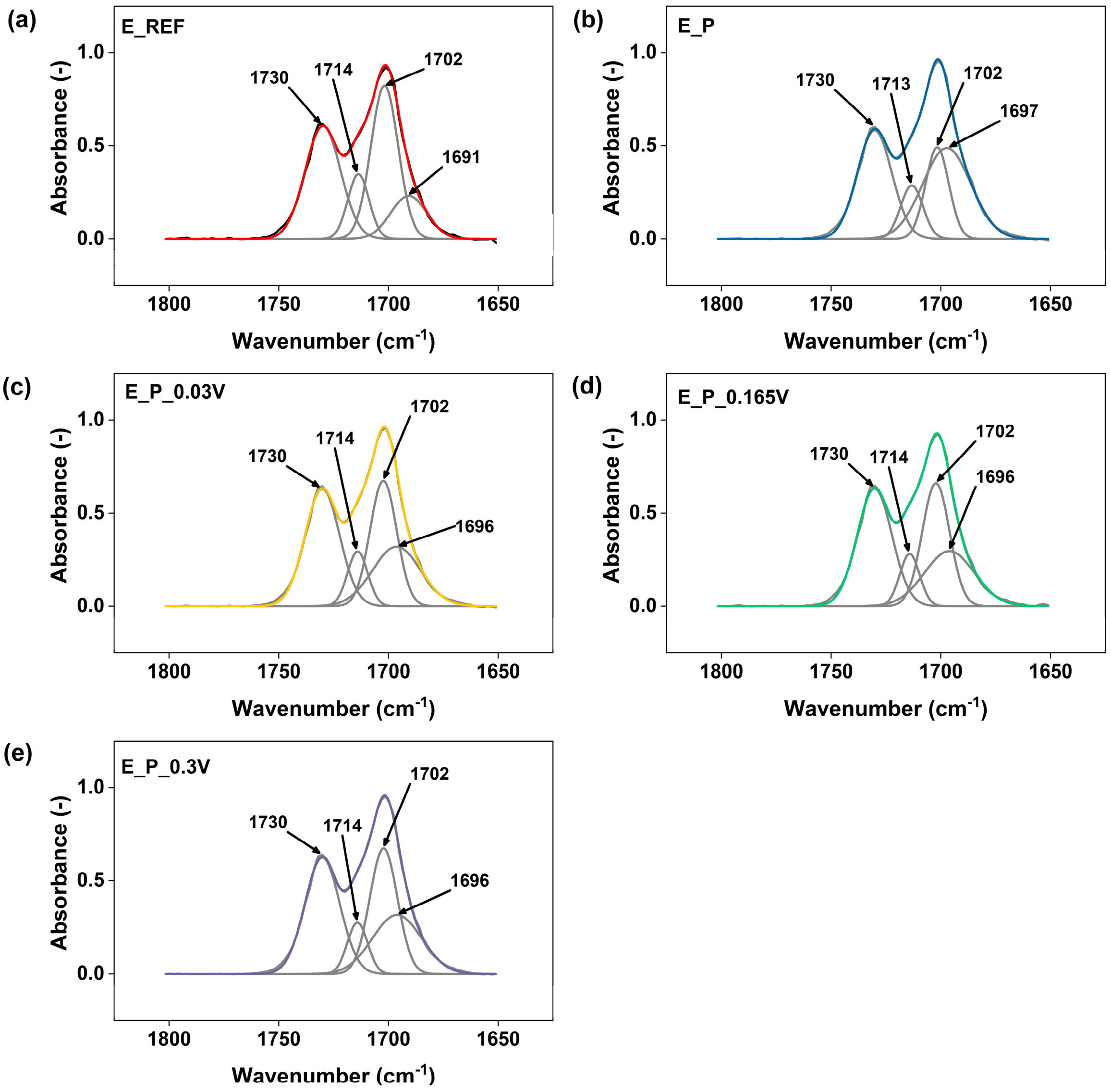

Figure S2. Deconvolution of the carbonyl band for E commercial TPU series for **(a)** E_REF, **(b)** E_P, **(c)** E_P_0.03V, **(d)** E_P_0.165V, **(e)** E_P_0.3V samples.

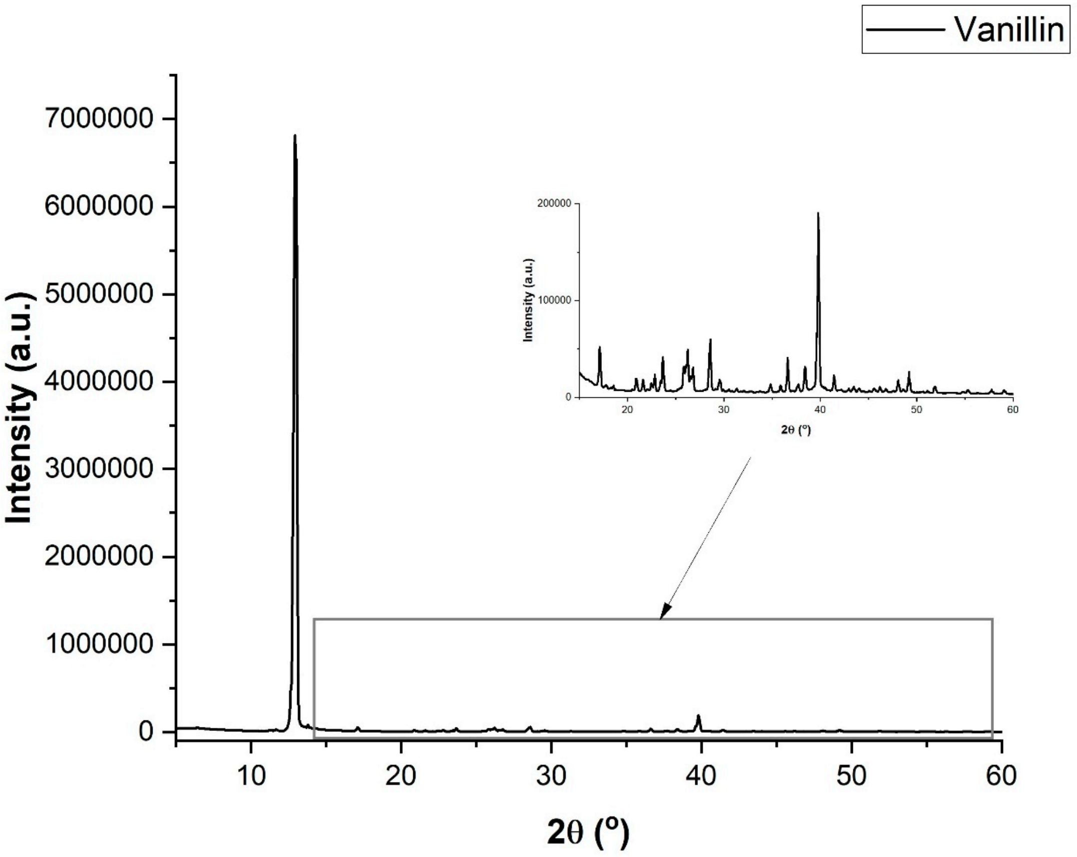

Figure S3. XRD pattern of vanillin.

Table S1. Photographs of the bio-TPUs and TPUs presented in the paper.

**HF_REF**	**E_REF**
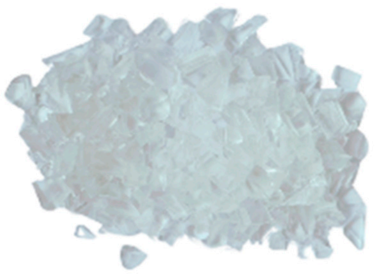	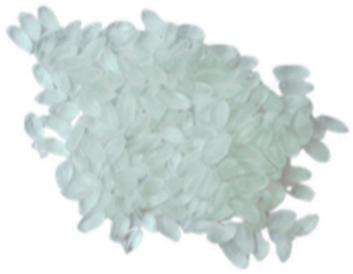

**HF_P** 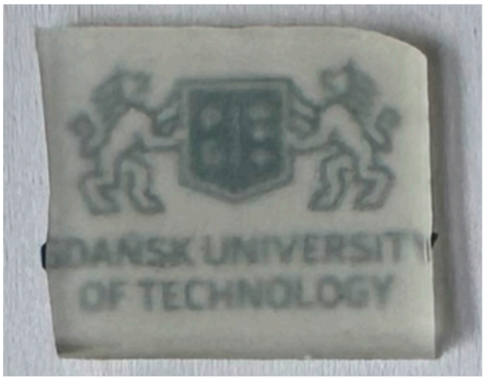	**E_P** 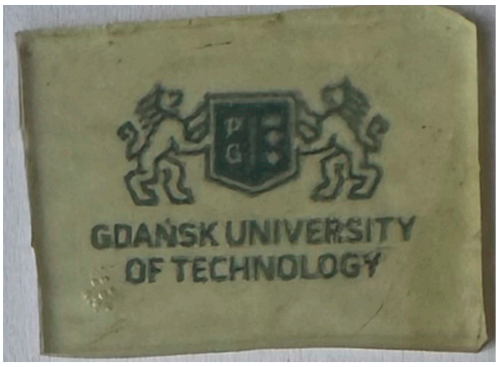
**HF_P_0.03V** 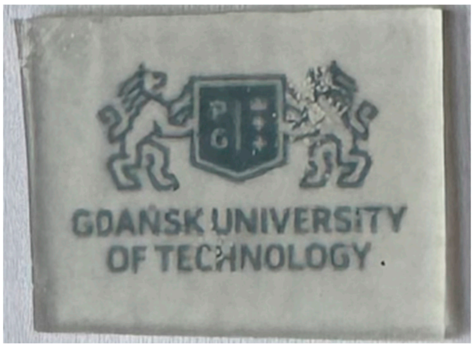	**E_P_0.03V** 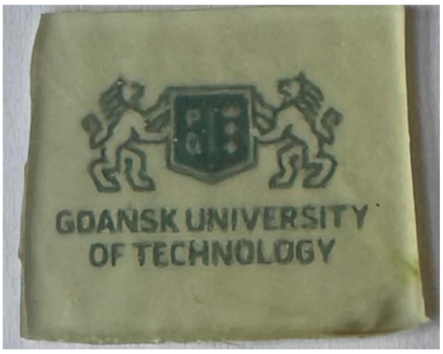
**HF_P_0.165V** 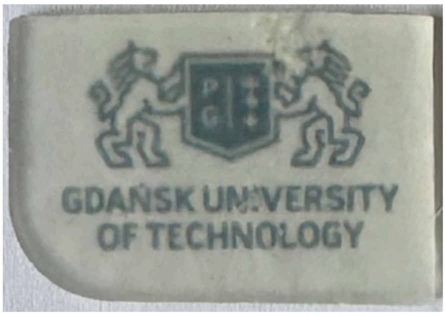	**E_P_0.165V** 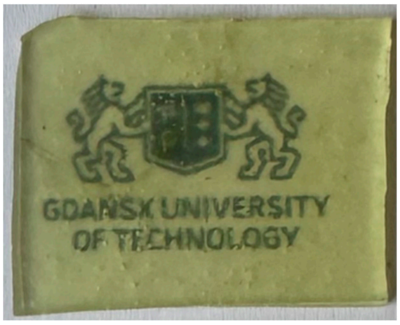
**HF_P_0.3V** 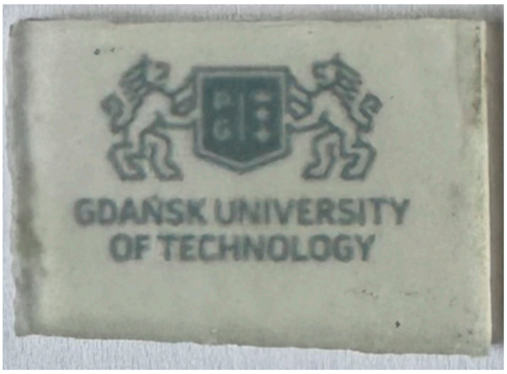	**E_P_0.3V** 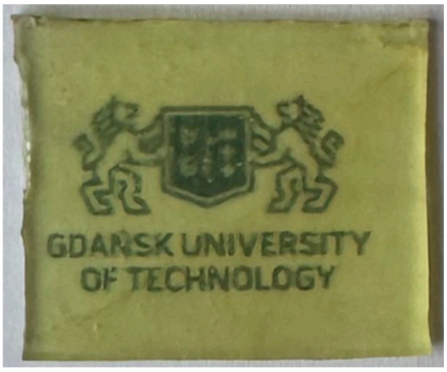

Table S2. Peak-fitting parameters (peak positions and maximum heights) and coefficient of determination (R^2) for the Gaussian components obtained from deconvolution of the carbonyl band (1600-1800 cm^{-1}), for HF and E TPU series.

Sample code	Peak position (cm^{-1})	Max Height	R^2
**HF_REF**	1683	0.64497	0.999
	1692	0.34113	
	1723	0.27367	
**HF_P**	1682	0.6247	0.998
	1689	0.34652	
	1722	0.22903	
**HF_P_0.03V**	1682	0.65581	0.998
	1689	0.32288	
	1722	0.22245	
**HF_P_0.165V**	1682	0.65932	0.998
	1689	0.33323	
	1722	0.2222	
**HF_P_0.3V**	1682	0.65721	0.998
	1690	0.32928	
	1722	0.21553	
**E_REF**	1691	0.23111	0.990
	1702	0.82247	
	1714	0.34841	
	1730	0.60655	
**E_P**	1697	0.48742	0.999
	1702	0.49108	
	1713	0.28709	
	1730	0.58365	
**E_P_0.03V**	1696	0.31972	0.999
	1702	0.67395	
	1714	0.29418	
	1730	0.63044	
**E_0.165V**	1696	0.29593	0.999
	1702	0.6609	

	1714	0.28115	
	1730	0.632	
**E_0.3V**	1696	0.31743	0.999
	1702	0.67378	
	1714	0.27543	
	1730	0.62546	

Table S3. The mean values of onset oxidation temperature (OOT) with standard deviations (n=3) and results of Tukey's post-hoc test for the HF and E TPU series. One-way ANOVA: HF series, $F(4,10) = 11.41$, $p = 0.0011$. E series, $F(4,10) = 548.71$, $p < 0.0001$. Samples sharing the same letter within a series do not significantly differ according to Tukey's post-hoc test ($p < 0.05$).

Sample code	OOT (°C)	Tukey group
**HF_REF**	174.9 ± 0.69	A
**HF_P**	167.9 ± 2.76	B
**HF_P_0.03V**	167.8 ± 2.00	B
**HF_P_0.165V**	173.3 ± 1.83	A
**HF_P_0.3V**	174.8 ± 1.74	A
**E_REF**	230.5 ± 1.54	A
**E_P**	174.2 ± 1.50	B
**E_P_0.03V**	226.6 ± 1.75	A
**E_P_0.165V**	230.8 ± 2.45	A
**E_P_0.3V**	228.1 ± 1.66	A
